# Molecular insights into Silodosin’s anti-cancer effects: a promising repurposing strategy for breast cancer

**DOI:** 10.1038/s41420-026-02973-8

**Published:** 2026-03-05

**Authors:** Michele Pellegrino, Maria Antonietta Occhiuzzi, Maria Marra, Francesca Giordano, Fedora Grande, Stefano Aquaro, Stefania Marsico, Paola Tucci

**Affiliations:** 1 Department of Pharmacy, Health and Nutritional Sciences, University of Calabria, Rende, Italy; 2 Department of Life, Health and Environmental Sciences, University of L’Aquila, L’Aquila, Italy

**Keywords:** Molecular biology, DNA damage and repair

## Abstract

Breast cancer, the most common cancer diagnosed among women worldwide, remains a significant clinical challenge due to its molecular heterogeneity and the development of resistance to conventional therapies. This study investigates the potential of silodosin, an α1A-adrenergic receptor (AR) antagonist, as a novel therapeutic agent in breast cancer, an area where its use has not been previously explored. Through detailed in vitro and in silico analyses, we elucidate the complex molecular mechanisms behind silodosin’s antitumor effects. We demonstrate its ability to inhibit cell proliferation, induce apoptosis, reduce migration, and prevent 3D spheroid formation. Importantly, these effects are observed across different breast cancer subtypes. Crucially, our integrated approach reveals that silodosin’s mechanism is not solely dependent on α1A-AR blockade. Molecular docking studies strongly suggest that silodosin directly interacts with both isoforms of the estrogen receptor (ERα and ERβ) with high affinity, binding to key residues within the ligand-binding domains of these receptors. This points to a new, dual-targeting mechanism of action. While its known α1A-AR antagonism may contribute to its effects, silodosin could also act as an ER ligand, modulating estrogen-driven pathways essential in breast cancer development. This study provides the first experimental and computational evidence supporting the repurposing of silodosin as a potential multi-targeted therapeutic agent for breast cancer, opening promising new opportunities for patients with limited treatment options and encouraging further preclinical and clinical studies.

## Introduction

Breast cancer remains one of the most frequently diagnosed malignancies and a leading cause of cancer-related mortality among women, representing a significant public health challenge. Epidemiological data from the World Health Organisation (WHO) highlight its considerable impact, with an estimated 2.3 million new cases and 685,000 deaths reported worldwide in 2020 alone [1]. Its profound molecular heterogeneity complicates the disease’s clinical management, manifesting as distinct subtypes with different biological behaviours and responses to therapy. While advances in early detection, molecular profiling, and targeted therapies have improved survival rates, significant challenges remain, particularly in cases of advanced or metastatic disease and among populations with limited access to healthcare.

The current classification of breast cancer is based on molecular profiling, which stratifies tumours into three main subtypes: (1) Hormone receptor-positive (HR+) tumours, which express estrogen (ER) and/or progesterone receptors (PR), comprise ~70% of cases and are typically managed with endocrine therapies (e.g., tamoxifen, aromatase inhibitors) [2]; (2) human epidermal growth factor receptor 2-positive (HER2+) tumours, driven by amplification/overexpression of the HER2 oncogene, account for 15–20% of cases and have seen significant improvements with HER2-targeted agents (e.g., trastuzumab, pertuzumab) [3]; (3) Triple-negative breast cancer (TNBC), characterized by the absence of ER, PR, and HER2 expression, is the most aggressive subtype, with limited treatment options and a poorer prognosis compared to other subtypes [4].

Recent years have seen notable progress in the development of targeted therapies and immunotherapies for breast cancer. Cyclin-dependent kinase 4/6 (CDK4/6) inhibitors, like palbociclib, ribociclib, and abemaciclib, when combined with endocrine therapy, have become the standard treatment for advanced HR+ breast cancer, significantly improving both progression-free survival and overall survival [5]. Antibody-drug conjugates like trastuzumab deruxtecan have demonstrated remarkable efficacy in refractory HER2+ breast cancer [6]. For TNBC, immunotherapy with checkpoint inhibitors, such as pembrolizumab, has shown benefits limited to a subset of patients, particularly those with programmed death-ligand 1 (PD-L1)-positive tumours [7]. Despite these advancements, significant challenges remain. Resistance to targeted therapies, particularly in HR+ and HER2+ breast cancers, is a considerable obstacle, necessitating the development of novel strategies to overcome or prevent resistance mechanisms [8]. Additionally, the aggressive nature of TNBC and its limited therapeutic options underscore the need for continued research into its biology and the identification of new therapeutic targets [9, 10].

In this context, Silodosin (SIL), a highly selective α1A-adrenergic receptor (AR) antagonist, has been widely recognized for its efficacy in treating lower urinary tract symptoms associated with benign prostatic hyperplasia (BPH). Its high selectivity for the α1A-AR subtype, predominantly expressed in the prostate and bladder neck, allows for targeted relief of urinary symptoms with minimal cardiovascular side effects [11–16]. Emerging evidence suggests that adrenergic signalling, mediated by α1-AR, may significantly influence cancer progression, including tumour growth, angiogenesis, and metastasis [17–19]. Stress-induced activation of the sympathetic nervous system and subsequent catecholamine release, such as norepinephrine and epinephrine, have been shown to promote tumour progression by stimulating ARs on cancer cells [20]. In particular, α1-ARs are involved in regulating cell proliferation, survival, and migration in various types of cancer, including prostate, bladder, pancreatic, and breast cancers [18, 21–23]. This has sparked interest in repurposing SIL beyond its urological applications, particularly in oncology. Due to its high selectivity for the α1A-AR, SIL offers a unique opportunity to investigate the therapeutic potential of adrenergic blockade in cancer. Although the application of SIL in oncology is still in its infancy, preliminary studies have shown promising results. For instance, in prostate cancer, SIL has been reported to inhibit cell proliferation and induce apoptosis in vitro, suggesting a potential role in slowing disease progression [24]. Similarly, in bladder cancer, SIL has demonstrated anti-tumour effects by reducing cell viability and migration [25]. These findings highlight the broader applicability of SIL in cancer therapy and underscore the need for further investigation into its mechanisms of action and therapeutic efficacy.

However, the potential for SIL to act beyond α1A-AR blockade is an area of growing interest. The concept of drug repurposing relies on identifying off-target effects that can be therapeutically exploited. Given the central role of ER signalling in the majority of breast cancers, any compound capable of modulating this pathway holds significant therapeutic potential. Interestingly, other α1-AR antagonists, such as doxazosin, have demonstrated anti-proliferative effects in breast cancer cells that appear to be independent of α1-AR expression, suggesting alternative mechanisms [13]. Furthermore, the MCF-7 cell line, a model for HR+ breast cancer, has been reported to lack substantial α1-AR expression [13], suggesting that any anti-cancer activity of SIL in these cells must involve other, non-adrenergic pathways.

Based on these observations, the present study aims to investigate the potential of SIL as a novel therapeutic agent in breast cancer, a field where its application has not yet been explored. We aim to examine the effects of SIL and further elucidate its mechanism of action in breast cancer cells in vitro, including both HR+ (MCF-7) and HR- (MDA-MB-231) cell lines. By repurposing SIL, we propose a new adjuvant therapeutic strategy to inhibit tumour growth and improve outcomes in breast cancer patients, particularly in TNBC, a subtype characterised by aggressive behaviour and limited treatment options.

## Results

### SIL reduces breast cancer cell growth

**Fig. 1:**
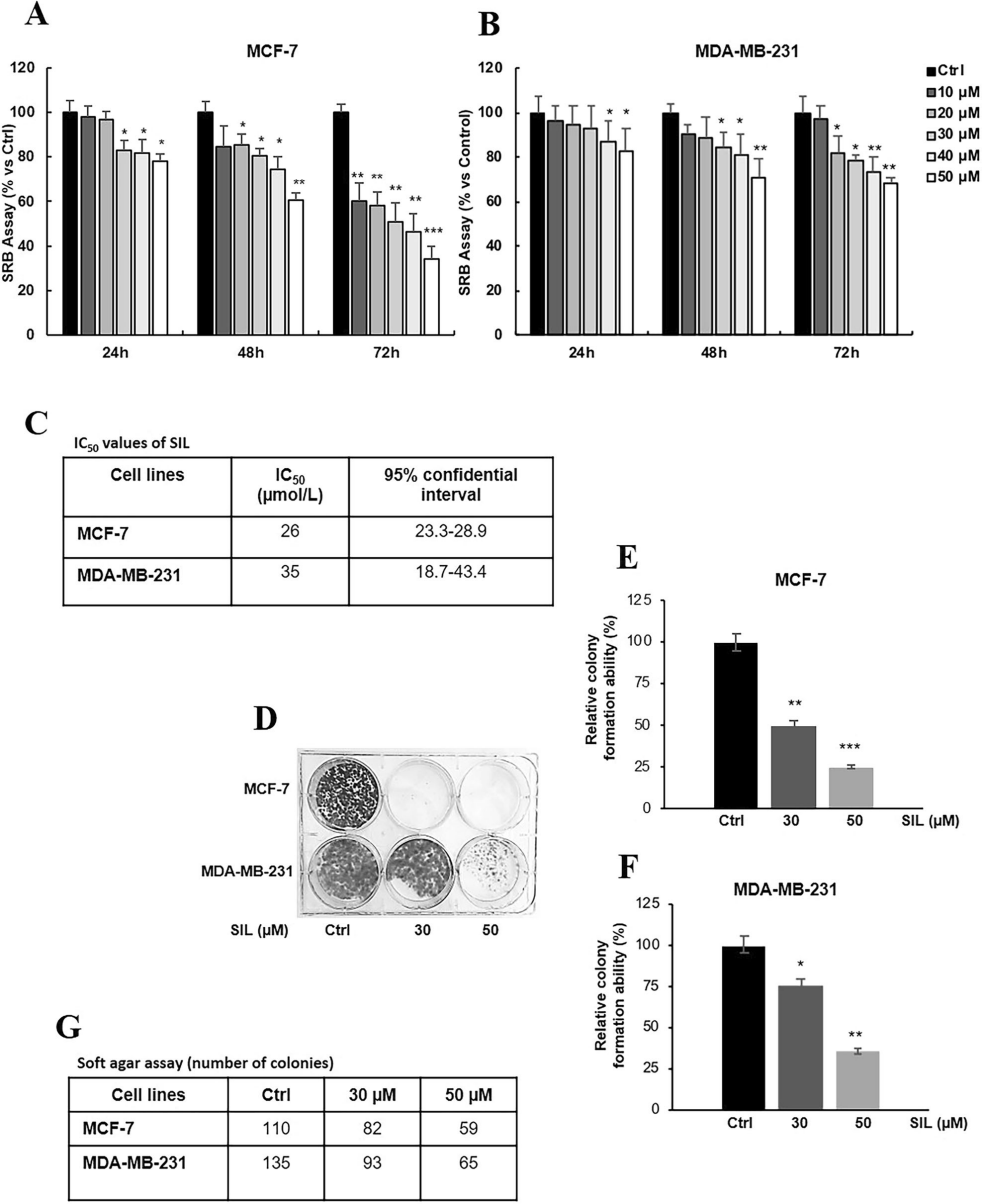
SIL inhibits breast cancer cell growth. Cells were treated with increasing concentrations (0, 10, 20, 30, 40, 50 μM) of SIL for 24, 48, and 72 hours, as indicated, and cell viability was assessed by SRB assay in **A** MCF-7 and **B** MDA-MB-231 cells. The results are expressed as % relative to control (Ctrl) cells. **C** SIL half-maximal inhibitory concentration (IC_50_) values for the indicated cells were calculated at 48 hours using GraphPad Prism 4 (GraphPad Software). **D**–**F** Clonogenic assay. MCF-7 and MDA-MB-231 cells were plated in 6-well plates and treated with SIL at concentrations of 30 and 50 μM, administered every three days. After 15 days, colonies were stained with crystal violet, photographed (**D**), and counted. The graphs show the % reduction in clonogenic ability of (**E**) MCF-7 and (**F**) MDA-MB-231 cells treated with SIL versus Ctrl. **(G)** Soft agar growth assay. Cells were plated in 0.35% agarose and treated as in (**D**). After three weeks, they were analysed and counted as the number of colonies. Data represent mean ± SD of three different experiments analysed in triplicate. *p < 0.05, **p < 0.01, ***p < 0.001.

We screened the antitumor potential of SIL across four breast cancer cell lines: MCF-7, MDA-MB-231, T47D, and MDA-MB-468. Each cell line is characterised by distinct expression profiles of estrogen receptor (ER), progesterone receptor (PR), and HER2. Additionally, we included the non-tumorigenic breast cell line MCF-10A as a control. As illustrated in Supplementary Fig. S1, SIL exhibited no significant inhibitory effect on the proliferation of normal MCF-10A cells. In contrast, its impact on tumour cell lines was time- and concentration-dependent, with sensitivity varying according to molecular subtype (Figs. 1A, B, and S2A, B). Notably, MCF-7 cells (Fig. 1A) and T47D cells (Figure S2A) were the most responsive, while triple-negative breast cancer (TNBC) cells, MDA-MB-231 (Fig. 1B) and MDA-MB-468 (Fig. S2B), demonstrated greater resistance to SIL. To provide an overview of SIL’s anti-proliferative effects, we included a graph comparing the cell survival curves across all tested cell lines at 24, 48, and 72 h for the concentrations of 30 and 50 µM of SIL, providing a clear visual representation of their differential sensitivity (Fig. S3A). After 48 h of treatment with 30 µM, MCF-7 proliferation was inhibited by 20%, and proliferative rates were further inhibited by 40% with 50 µM. The half-maximal inhibitory concentration (IC_50_) values for SIL in MCF-7 (26 µmol/L) and MDA-MB-231 (35 µmol/L) are summarized in Fig. 1C. Furthermore, the corresponding IC₅₀ values for all cancer cell lines, including T47D and MDA-MB-468, are outlined in Fig. S3B.

To further validate these findings, we performed clonogenic assays, treating the cells with two concentrations of SIL (30 µM and 50 µM), selected based on prior antiproliferative data, to compare the sensitivity among the different cell lines. SIL treatment markedly suppressed colony formation in both MCF-7 (Fig. 1E) and MDA-MB-231 (Fig. 1F) cells under both anchorage-dependent (clonogenic assay, Fig. 1D) and anchorage-independent (soft agar assay, Fig. 1G) conditions. Specifically, prolonged exposure (up to 15 days) effectively impeded clonogenic growth in both cell lines, including the more resistant TNBC model MDA-MB-231, albeit with reduced efficacy compared to MCF-7. The combined use of these assays confirms that SIL exerts an antiproliferative effect under both monolayer conditions (clonogenic assay) and in a more tumour-like context (soft agar). Moreover, these findings indicate that SIL possesses significant antiproliferative activity against breast cancer cells, regardless of their HR status, highlighting its potential as a therapeutic agent for breast cancer, even in aggressive subtypes like TNBC.

### Effect of SIL on cell cycle and apoptosis

**Fig. 2:**
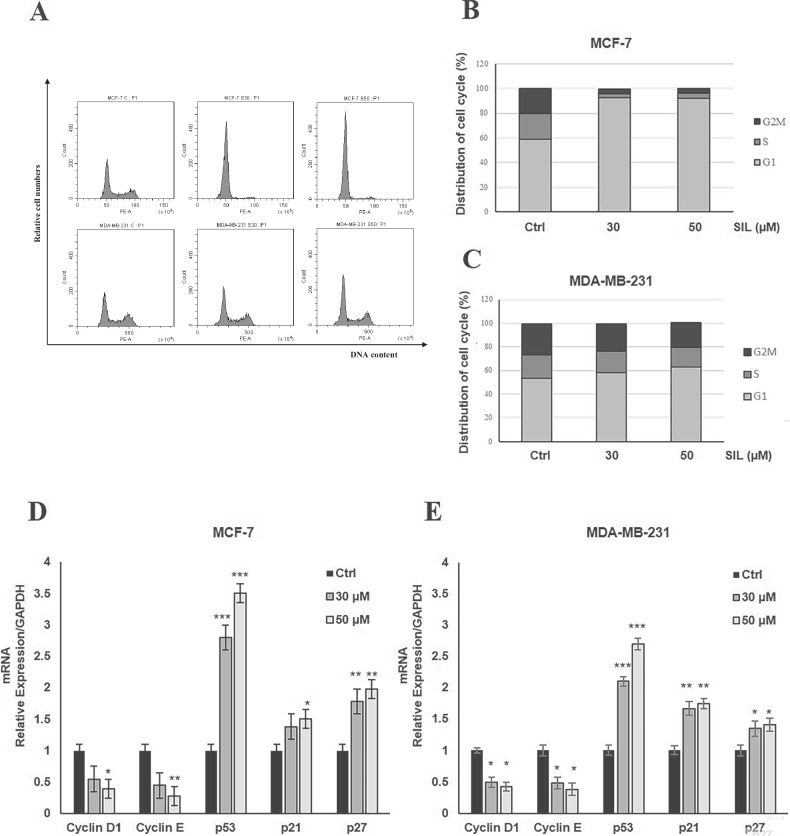
SIL inhibits cell-cycle progression. **A** Cells were treated with SIL 30 and 50 μM, and cell cycle analysis was performed by flow cytometry after 48 h of treatment in **B** MCF-7 and **C** MDA-MB-231 cells. The expression of cell-cycle–related genes (cyclin D1, cyclin E, p53, p21, and p27) was evaluated by qRT-PCR after 24 h of treatment in **D** MCF-7 and **E** MDA-MB-231 cells. The qRT-PCR results were normalized to the GAPDH gene. Data represent mean ± SD of three different experiments analysed in triplicate. *p < 0.05, **p < 0.01, ***p < 0.001.

To investigate whether the observed inhibition of cell growth induced by SIL was linked to alterations in the cell cycle, we performed flow cytometric cell-cycle analysis in the selected breast cancer cell lines following 48 h of treatment with SIL at concentrations of 30 and 50 µM. The results demonstrated that SIL induced a significant cell-cycle arrest in the G0–G1 phase, accompanied by a concurrent decrease in the proportion of cells in the G2–M phase. This effect was more pronounced in MCF-7 cells (Fig. 2A, B) compared to MDA-MB-231 cells (Fig. 2A, C), where the response was still detectable, although less robust.

**Fig. 3:**
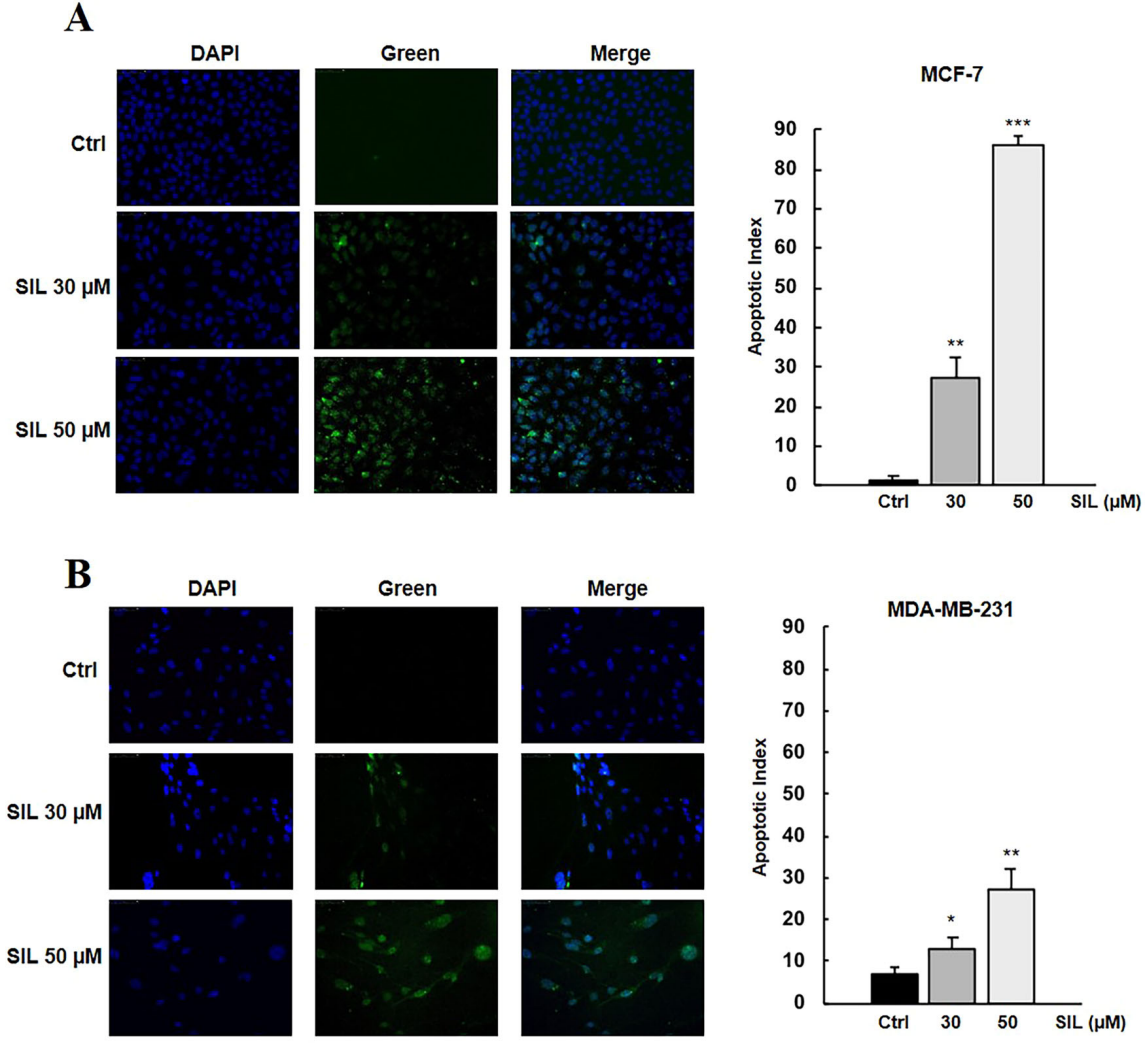
SIL induces apoptosis. Cells were treated with SIL 30 and 50 μM, and apoptosis was assessed by TUNEL assay in **A** MCF-7 and **B** MDA-MB-231 cells. Apoptotic cells were imaged at 20X magnification using an Olympus DP50 camera and ViewFinder software, and the apoptotic index was quantified using ImageJ software. *p < 0.05, **p < 0.01, ***p < 0.001.

Additionally, we analysed the gene expression levels of key proteins involved in cell-cycle regulation using qRT-PCR. Consistent with the observed cell-cycle arrest, SIL treatment for 24 h led to a downregulation of cyclin D1 and cyclin E, both critical regulators of G1–S phase transition, in both MCF-7 (Fig. 2D) and MDA-MB-231 (Fig. 2E) cell lines. Conversely, the expression of the tumour suppressor p53 and the cell-cycle inhibitors p21 and p27 was significantly upregulated in both cell lines (Fig. 2D, E). To better understand the molecular mechanisms underlying the antiproliferative effects of SIL, we evaluated its antitumor potential by assessing its ability to induce apoptosis using the TUNEL assay. Our findings confirmed that after 72 h of treatment, SIL exerts a pro-apoptotic effect in a dose-dependent manner, as evidenced by an increase in apoptotic cells in both tested cell lines (Fig. 3A, B).

These results support the hypothesis that SIL modulates cell-cycle progression and promotes apoptosis, reinforcing its potential as an antitumor agent.

### SIL inhibits cell migration

**Fig. 4:**
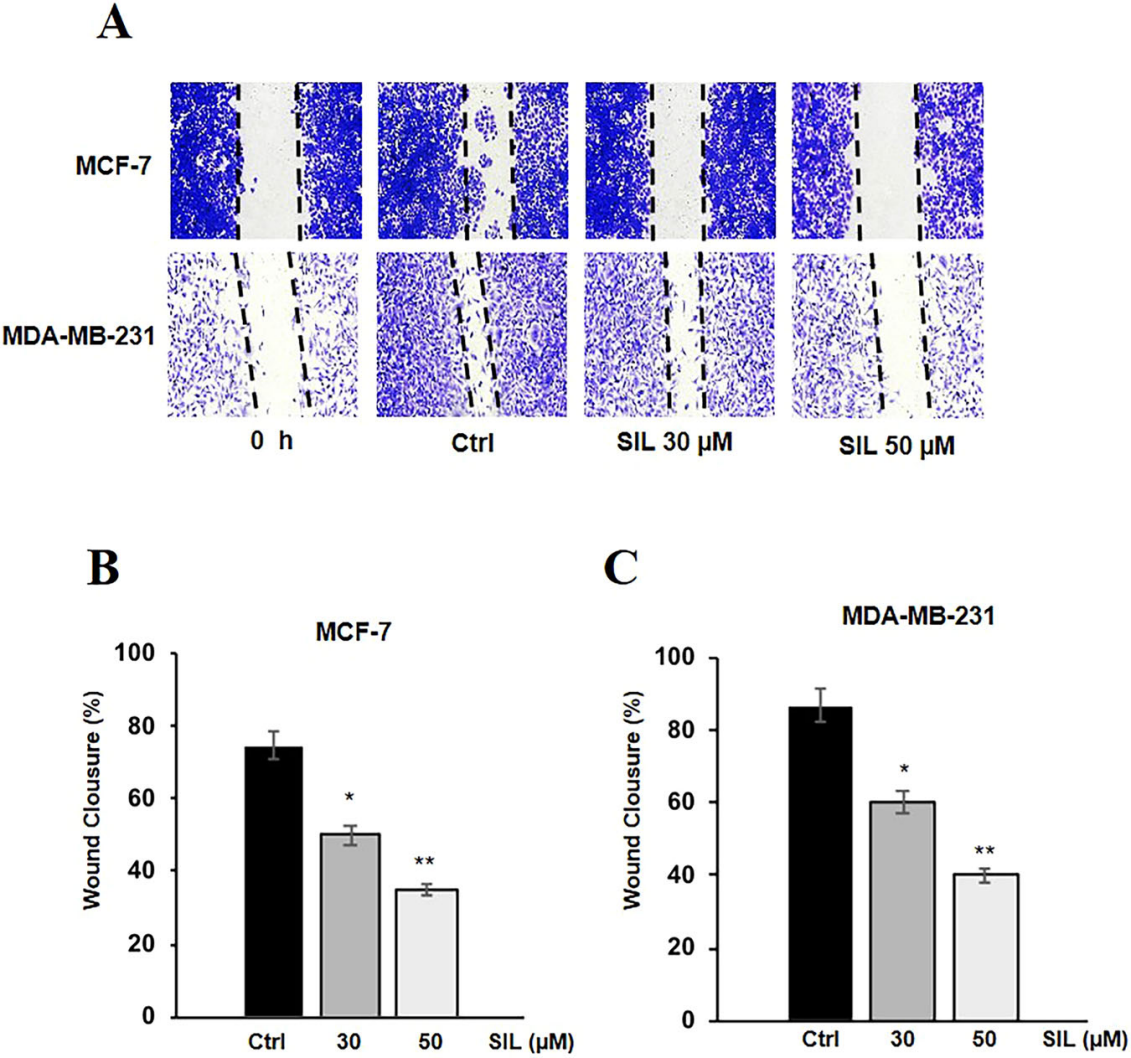
SIL inhibits cell migration. **A** Wound healing (scratch) assays of **B** MCF-7 and **C** MDA-MB-231 cells treated with SIL 30 and 50 μM for 48 hours. Monolayers were scratch-wounded with a p10 tip and observed under microscopy. The wound closure is illustrated **A** by showing the wound immediately (0 h) and 48 h after the scratch. Images are representative of eight fields from three independent experiments. Data represent mean ± SD of three different experiments analysed in triplicate. *p < 0.05, **p < 0.01.

To investigate the potential anti-metastatic effects of SIL, we examined its impact on cell migration using wound-healing (scratch) assays in the breast cancer cell lines (MCF-7 and MDA-MB-231). Treatment with SIL (30 and 50 µM) significantly inhibited wound closure, in a dose-dependent manner, in both MCF-7 (Fig. 4A, B) and MDA-MB-231 (Fig. 4A, C) cells, with approximately 70% and 60% decrease, respectively, after 48 h of treatment at 50 µM.

### SIL prevents the spheroid formation in breast cancer cell lines

**Fig. 5:**
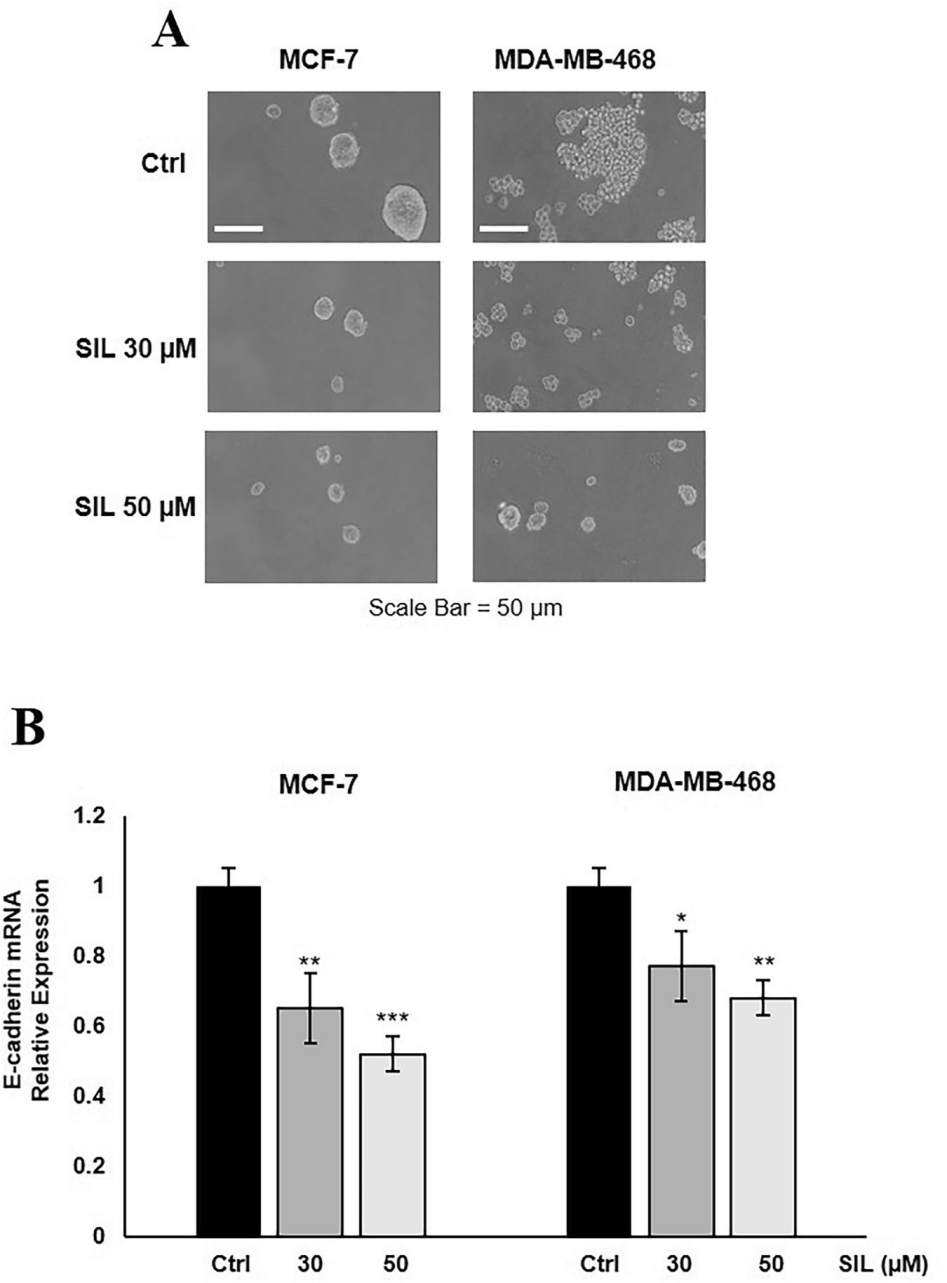
SIL inhibits 3D aggregation. MCF-7 and MDA-MB-231 cells were treated with SIL at 30 and 50 μM, and the formation of 3D spheroids was observed using a phase-contrast microscope (Olympus, Milan, Italy). **A** Representative 3D culture. **B** The expression levels of E-cadherin were evaluated by qRT-PCR in 3D cultures of MCF-7 and MDA-MB-468 cells. The qRT-PCR results were normalized to the GAPDH gene. Data represent mean ± SD of three different experiments analysed in triplicate. *p < 0.05, **p < 0.01, ***p < 0.001.

Multicellular tumour spheroids have become a gold-standard model in oncology research due to their ability to mimic key aspects of solid tumours. Unlike traditional 2D monolayer cultures, these three-dimensional (3D) structures recapitulate critical tumour features, including nutrient gradients, hypoxia, and cell-cell interactions. They can simulate avascular tumour areas comprising proliferative and necrotic cells. To evaluate the anti-tumour potential of SIL in this more physiologically relevant context, we assessed its ability to prevent 3D spheroid formation under low-attachment conditions using MCF-7 and MDA-MB-468 breast cancer cell lines. We observed that both tested concentrations of SIL effectively blocked spheroid formation across all cell lines (Fig. 5A and Table 1). Both cells showed great susceptibility to SIL’s effects, exhibiting near-complete inhibition of spheroid formation at the highest concentration. The inhibitory activity was dose-dependent, with higher concentrations causing more pronounced effects, including a reduction in spheroid size. Inhibition of spheroid formation suggests that SIL may interfere with critical pathways required for 3D tumour organisation, such as survival signalling or cell aggregation and adhesion mechanisms (e.g., E-cadherin-mediated interactions). Thus, to explore the role of cell adhesion in inhibitory effects of SIL, we analysed the expression levels of E-cadherin via qRT-PCR and found that SIL treatment significantly downregulated E-cadherin expression levels (Fig. 5B).

**Table 1:** Effect of SIL on cell aggregation in MCF-7 and MDA-MB-468 breast cancer cells.

MCF-7	SPHEROIDS		
	25 ≤ 50 µm	50 ≤ 100 µm	˃100 µm
**Ctrl**	5 ± 1.3	42 ± 2.7	77 ± 2.3
**SIL 30** **µM**	53 ± 1.8	35 ± 2.2	4 ± 2.5
**SIL 50** **µM**	81 ± 2.9	11 ± 0.7	0.0 ± 0.0
**MDA-MB-468**	**SPHEROIDS**		
	**25** ≤ **50** **µm**	**50** ≤ **100** **µm**	**˃100** **µm**
**Ctrl**	3 ± 1.7	37 ± 2.2	82 ± 1.6
**SIL 30** **µM**	59 ± 1.3	21 ± 2.7	3 ± 1.8
**SIL 50** **µM**	83 ± 1.3	9 ± 2.7	0.0 ± 0.0

MCF-7 and MDA-MB-468 cells were cultured as 3D spheroids in complete growth medium. The extent of aggregation was scored by measuring the spheroid diameters with an ocular micrometre. The values represent a sum of 3D cultures in 10 optical fields under 10X magnification. The results are mean ± SD from at least three experiments. Representative 3D cultures are shown in Fig. 5A.

These findings suggest that the ability of SIL to block 3D tumour spheroid formation is mediated, at least in part, by modulating adhesion molecules.

### In silico molecular docking reveals SIL as a potential ER ligand

SIL exerts potent anti-proliferative and pro-apoptotic effects in MCF-7 cells, a model that lacks functional α1A-adrenergic receptor (α1A-AR) expression [18]. This provides indirect evidence that in these cells, the drug’s mechanism must be independent of α1-AR blockade. In the literature, there are relatively few articles that determine the expression of all ARs in breast cancer cells. Thus, we evaluated the expression levels of ADRA1A (the gene encoding the α1A-AR) in all the breast cancer cell lines used, compared to the non-tumorigenic MCF-10A cell line, by qRT-PCR (Fig. S4). This analysis confirmed the reported lack of α1A-AR expression in MCF-7 cells. Interestingly, it also shows variable expression levels in the other lines (T47D, MDA-MB-231, MDA-MB-468). Crucially, there is no clear correlation between the level of α1A-AR expression and sensitivity to SIL (as measured by our IC₅₀ values). For instance, T47D cells, which express α1A-AR, are sensitive, whereas MDA-MB-231 and MDA-MB-468 cells, which also express α1A-AR, are more resistant. This lack of correlation suggests that the anti-cancer effects of SIL are not primarily mediated through the α1A-AR.

Given that the anti-proliferative effects of SIL were particularly potent in ER + MCF-7 cells and that our data showed no correlation between α1A-AR expression and efficacy, we hypothesized that SIL might directly interact with ER. To test this, a structure-based molecular docking study was performed using two selected crystallographic structures of ERα and ERβ retrieved from the PDB (entry codes 3ERT and 2FSZ for ERα and ERβ, respectively) [26] and 2FSZ for Erβ [27]. Both receptors in these structures are complexed with 4-hydroxytamoxifen (OHTX), an active metabolite of tamoxifen, which is known as a high-affinity, selective estrogen receptor modulator (SERM). ERβ contains an additional surface-exposed allosteric site (site 2) in which a second molecule of OHTX is bound. As a first step of our simulation procedure, re-docking experiments of the crystallographic ligand into the ERα binding site, and for ERβ in sites 1 and 2, were performed to assess the reliability of the docking protocol and establish reference binding affinities. As a result, binding energy (BE) values of −8.8, −8.1, and −5.5 kcal/mol for ERα and ERβ site 1 and site 2, respectively, were obtained. These results confirmed the expected preference of the ligand for the canonical binding sites over the secondary site and demonstrated correct pose reproduction within each binding domain. To provide additional thermodynamic reference values beyond BE, the inhibition constant (Ki) and ligand efficiency (LE) for the re-docked ligand in all three sites were calculated leading to Ki values of 0.63, 1.96 and 134 μM for ERα, ERβ site, and ERβ site 2, respectively, while the corresponding LE values were −0.30, −0.28, and −0.19 kcal/mol per heavy atom, respectively. These values, consistent with the BE values, further validated the accuracy and reliability of the docking protocol and were taken as reference. Following re-docking validation, SIL was docked into the same three binding pockets, and the results revealed distinct binding profiles across the receptor subtypes and sites. When docked in the ERα binding site, SIL showed a favourable binding energy of −7.8 kcal/mol. To further quantify this affinity, the Ki and LE were calculated, yielding values of 3.22 μM and −0.22 kcal/mol per heavy atom, respectively (Table 2).

**Table 2:** Binding energy (BE), inhibition constant (Ki), and ligand efficiency (LE) values for OHTX and Silodosin (SIL), along with key protein residues interacting with each ligand within the ERα binding site.

LIGAND	CHEMICAL STRUCTURE	BE kcal/mol	Ki μM	LE kcal/mol	INTERACTIONS				
					Hydrogen Bonds				Hydrophobic Interactions Residues
					Residues	Distance Å		Donar Angle°	
						H-A	D-A		
**OHTX**		-8.8	0.63	-0.30	Glu353 Arg394	1.61 2.09	2.42 3.03	142.53 157.43	Leu346, Ala350, Trp383, Leu384, Leu387, Phe404, Leu428, Leu525
**SIL**		−7.8	3.22	-0.22	Leu387	2.36	3.13	136.69	Ala350, Trp383, Leu384, Leu387, Leu391, Phe404, Leu525

**Fig. 6:**
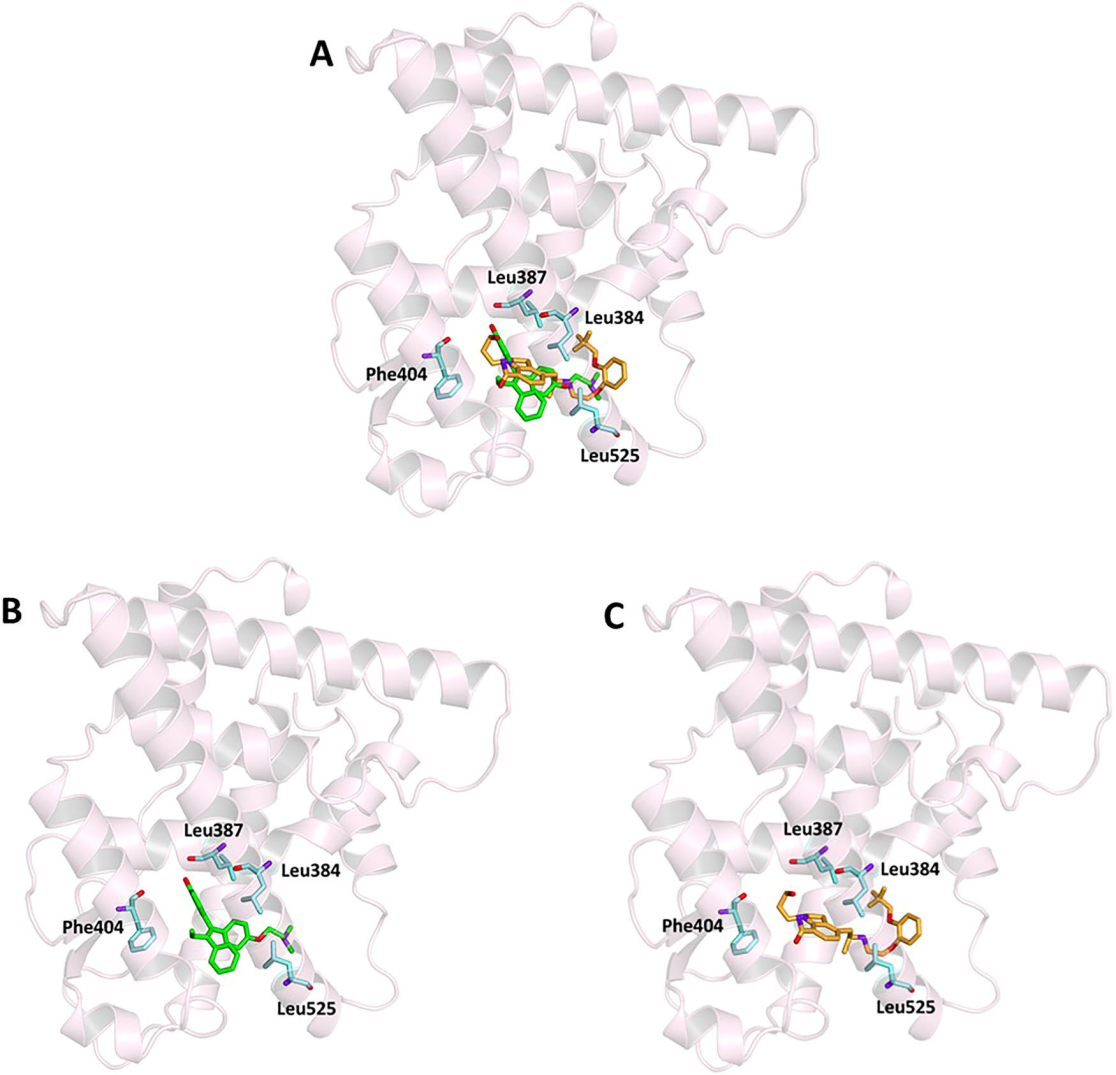
Ligand-binding pocket of the active site of ERα. The protein backbone (PDB code: 3ERT) is represented in background as ribbons, and key protein residues are highlighted in aquamarine. **A** Superimposed binding modes of OHTX (green) and SIL (orange). The ligands are also shown separately: **B** OHTX and **C** SIL.

These results, despite being slightly lower than those of OHTX, remain consistent with a biologically meaningful interaction and reinforce the validity of the docking predictions. SIL is well fitted within the receptor binding pocket, similarly to OHTX (Fig. 6), interacting through a hydrogen bond with Leu387 mediated by its hydroxyl group, and several hydrophobic interactions involving residues such as Ala50, Trp383, Leu384, Phe404, and Leu525, also involved in the interaction of the crystallographic ligand. The presence of shared interacting residues with OHTX suggests that SIL adopts a compatible binding mode within the ERα ligand-binding domain, potentially allowing for functional modulation (Fig. 6 and Table 2).

**Fig. 7:**
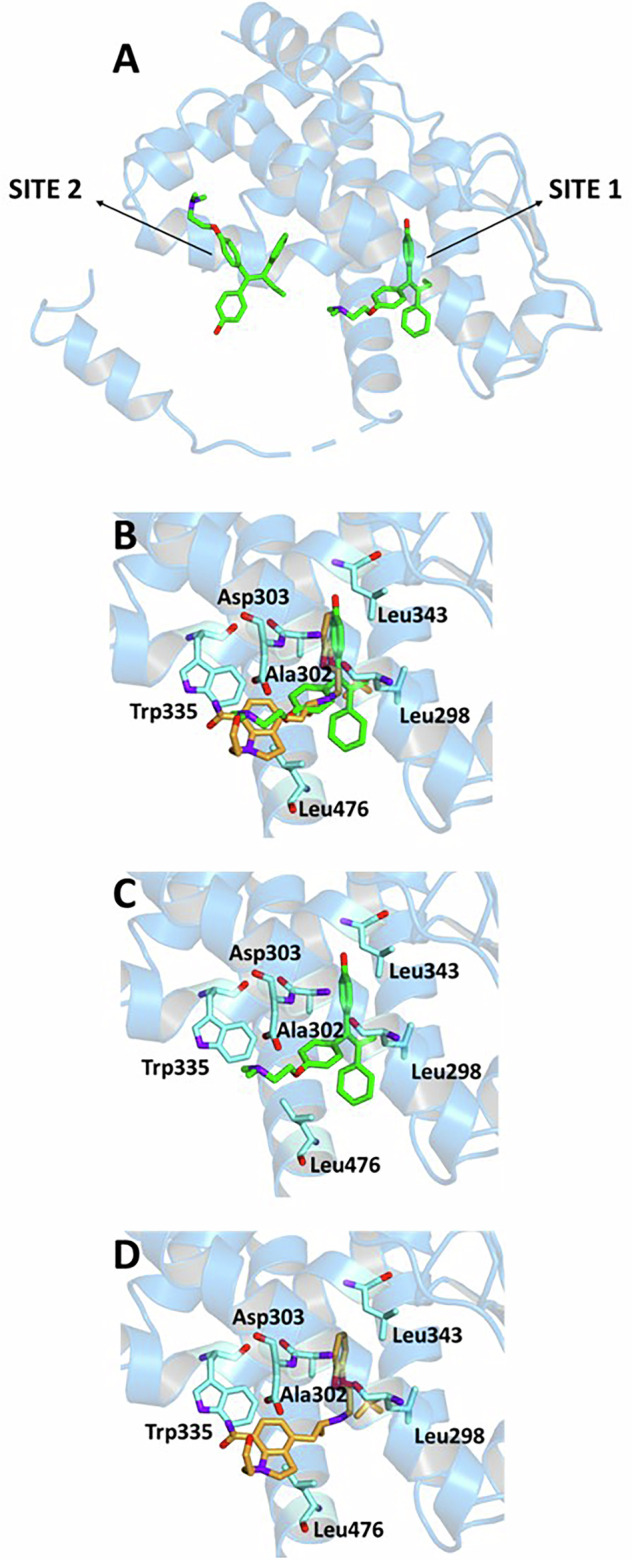
Ligand-binding pockets of ER (PDB code: 2FSZ). The protein backbone is represented in background as ribbons, and key protein residues are highlighted in aquamarine. **A** OHTX (green) bound at sites 1 and 2. **B** Superimposed binding conformations of OHTX (green) and SIL (orange) at site 1. Individual ligands at site 1 are also shown separately: **C** OHTX and **D** SIL.

Site-specific docking simulations at the primary ligand-binding site of ERβ (site 1) revealed that SIL adopts a stable conformation within the pocket (Fig. 7), with a BE value of −7.4 kcal/mol. This result, although slightly less favourable than the value observed for its interaction with ERα, still indicates an interaction of potential pharmacological significance. The corresponding Ki was estimated at 6.1 μM, and the LE at −0.21 kcal/mol per heavy atom, both of which are consistent with the predicted BE and reflect a good binding affinity (Table 3). The SIL-ERβ site 1 complex is characterized by the formation of three hydrogen bonds with key residues Leu298, Thr299, and Asp303 of the receptor, which contribute significantly to ligand anchoring within the site. In addition to these polar interactions, the complex is further stabilized by a network of hydrophobic contacts, with several residues including Ala302, Trp335, Leu339, Leu343, and Leu476, which are also involved in the interaction profile of the co-crystallized ligand OHTX, suggesting a partial overlap in binding mode and reinforcing the hypothesis that ERβ site 1 represents a viable target for SIL.

**Table 3:** Binding energy (BE), inhibition constant (Ki), and ligand efficiency (LE) values for OHTX and Silodosin (SIL), along with key protein residues interacting with each ligand within the ERα binding site 1.

LIGAND	BE kcal/mol	Ki μM	LE kcal/mol	INTERACTIONS					
				Hydrogen Bonds				Hydrophobic Interactions Residues	Salt Bridges
				Residues	Distance Å		Donar Angle °		
					H-A	D-A			
**OHTX**	−8.1	1.96	−0.28	Glu305 Arg346	2.00 1.92	2.67 2.73	127.21 138.32	Leu298, Leu301, Ala302, Trp335, Leu343, Phe356, Ile373, Ile376, Leu380, Leu476	Asp303
**SIL**	−7.4	6.1	−0.21	Leu298 Thr299 Asp303 Asp303	2.67 3.47 2.47 2.54	3.59 3.82 3.42 2.88	150.57 103.44 163.40 100.46	Thr299, Ala302, Leu306, Trp335, Leu339, Leu343, Leu476	

**Fig. 8:**
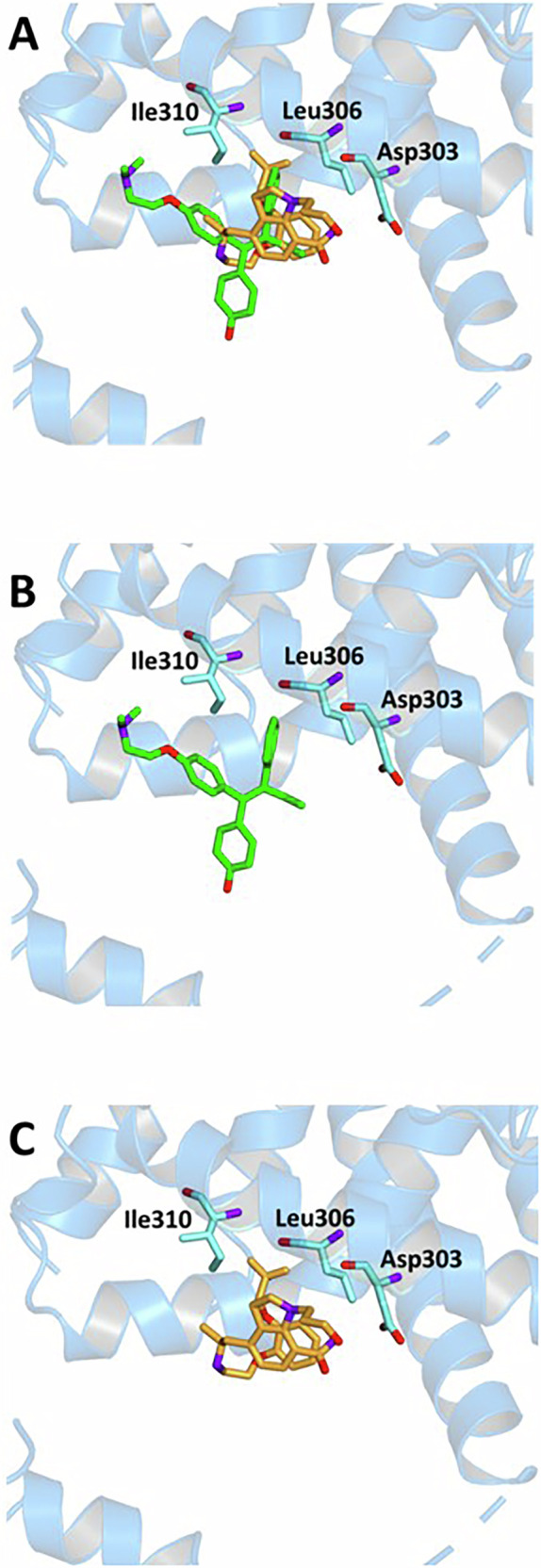
Ligand-binding pocket of the secondary site of ERβ (site 2). The protein backbone is represented in background as ribbons, and key protein residues are highlighted in aquamarine. **A** Superimposed binding modes of OHTX (green) and SIL (orange) in site 2. The ligands are also shown separately: **B** OHTX and **C** SIL.

Previous structural investigations have identified a second binding pocket (site 2) on the outer surface of ERβ, distinct from the canonical ligand-binding domain. Although this site is structurally more exposed and less conserved, it is believed to play an allosteric role in the fine regulation of receptor activity. As such, ligands capable of binding to this region may contribute to a complementary modulation of estrogen signalling. Accordingly, we also docked SIL at the ERβ site 2. As expected for an allosteric interaction, SIL showed a lower affinity (BE = −5.9 kcal/mol) compared to that recorded for the primary binding site. The calculated Ki and LE values were also consistent with a weaker, yet potentially modulatory, interaction (Ki = 69.8 μM and LE = −0.17 kcal/mol). Specifically, SIL formed a single hydrogen bond with Asp303, along with hydrophobic interactions with residues including Leu306, Val307, Ile310, and Trp335. Also, in this case, some of these residues are involved in the crystallographic ligand interaction. These findings suggest that, while SIL does not form a tightly bound complex at site 2, its ability to interact with this region may still hold functional relevance. The observed binding parameters are consistent with the expected behaviour of ligands at secondary pockets and highlight the importance of considering site-specific receptor-ligand interactions (Fig. 8 and Table 4).

**Table 4:** Binding energy (BE), inhibition constant (Ki), and ligand efficiency (LE) values for OHTX and Silodosin (SIL), along with key protein residues interacting with each ligand within the ERβ binding site 2.

LIGAND	BE Kcal/mol	Ki μM	LE kcal/mol	INTERACTIONS				
				Hydrogen Bonds				Hydrophobic interactions residues
				Residues	Distance Å		Donar Angle °	
					H-A	D-A		
**OHTX**	−5.5	134	−0.19					Leu306, Ile310, Val328, Leu331, Glu332, Trp335
**SIL**	−5.9	69.8	−0.17	Asp303 Asp303	2.35 3.28	3.22 3.80	147.09 115.54	Leu306, Val307, Ile310, Trp335

## Discussion

Breast cancer is a heterogeneous disease, with clinical outcomes and responses to therapy varying significantly depending on the molecular subtypes. Triple-negative breast cancer (TNBC), which accounts for approximately 10–15% of all breast cancer cases, is characterised by an aggressive phenotype, high genomic instability, a strong tendency to develop metastases, and resistance to chemotherapy. Although taxane-based chemotherapy remains the standard treatment for most early and advanced TNBC cases, clinical outcomes are still poor compared to other subtypes. Patients with TNBC face a high risk of relapse and exhibit lower survival rates, particularly in metastatic settings. As a result, there is an urgent need to identify and develop new molecularly targeted therapies to improve treatment outcomes for TNBC.

SIL is a novel α1-AR antagonist that is selective for the α1A-AR. The US Food and Drug Administration (FDA) approved it in 2008 for the treatment of lower urinary tract symptoms (LUTS) associated with BPH [11–16].

This study aimed to evaluate the potential antitumor effects of SIL in breast cancer, a field where its application has not been previously explored, and to clarify its mechanism of action beyond α1A-AR blockade.

Notably, SIL exhibited selective cytotoxicity, sparing normal breast epithelial cells (MCF-10A) while significantly suppressing proliferation in all tested cancer cell lines, albeit with variable efficacy. The differential sensitivity observed among cell lines of the same subtype (e.g., MCF-7 vs. T47D; MDA-MB-231 vs. MDA-MB-468) likely reflects the vast genetic and phenotypic heterogeneity of breast cancer. For instance, while both are HR+, T47D cells harbour PIK3CA and TP53 mutations, potentially altering their dependency on specific survival pathways compared to MCF-7. Similarly, within TNBC, MDA-MB-468 cells are PTEN-null, while MDA-MB-231 cells are characterized by KRAS mutations and a highly mesenchymal phenotype. These distinct oncogenic drivers likely account for their different responses to SIL and underscore the complexity of predicting drug efficacy based solely on receptor status.

Nevertheless, its ability to inhibit even resistant TNBC cells in clonogenic and soft agar assays underscores its potential applicability across heterogeneous breast cancers.

The antiproliferative effects of SIL were mechanistically linked to G0/G1 cell cycle arrest, mediated by downregulation of cyclin D1 and cyclin E, and concomitant upregulation of p53, p21, and p27. This aligns with the known roles of these regulators in restraining G1-S progression and highlights SIL’s capacity to reactivate tumour-suppressive checkpoints. Furthermore, SIL-induced apoptosis, as evidenced by TUNEL assays, reinforces its dual cytostatic and cytotoxic actions. The partial resistance of MDA-MB-231 cells to these effects may reflect their basal-like/TNBC phenotype, characterised by inherent apoptotic evasion; however, prolonged SIL exposure still impaired their clonogenicity, suggesting that alternative mechanisms (e.g., senescence or metabolic disruption) could contribute to its efficacy. This subtype-agnostic activity is particularly significant given the limited therapeutic options for patients with HR- and TNBC.

Therefore, we demonstrated that SIL exerts potent antiproliferative and pro-apoptotic effects across distinct breast cancer subtypes, including hormone receptor (HR)-positive MCF-7 and triple-negative MDA-MB-231 cells.

Crucially, our data suggest that the anti-tumour effects of SIL are not solely dependent on α1-AR signalling, indicating additional molecular targets that depend on the context. This is supported by two key findings: firstly, the strong activity observed in MCF-7 cells, which we have shown lacks α1A-AR expression, and secondly, the absence of a correlation between α1A-AR mRNA levels and SIL sensitivity across all cell lines.

The in silico docking studies provide a compelling and novel mechanistic insight: SIL preferentially binds to ERα and the main (canonical) binding site of ERβ (site 1), with favourable binding energies, low Ki, and good LE values consistent with meaningful and potentially functional molecular interactions. Notably, in both of these sites, SIL interacts with several key residues also engaged by OHTX, supporting a plausible mechanism of receptor recognition and suggesting potential structural mimicry of known selective ER modulators (SERMs). In contrast, the less favourable binding energy, higher Ki, and lower LE observed in site 2 of ERβ suggest a limited contribution of this site to SIL’s biological activity. This reduced performance may reflect not only the site’s structural characteristics—such as its shallow and solvent-exposed topology—but also its distinct functional role as an allosteric or secondary regulatory site, which may not be optimized for high-affinity ligand binding in the same way as the canonical domain.

These findings support our biological results, strengthening the hypothesis that SIL may act as a modulator of ER function. In ER+ breast cancers, such as MCF-7, this interaction could potentially antagonize or modulate the proliferative signals driven by estrogen, leading to the observed downregulation of cyclin D1 and cell cycle arrest. The binding to ERβ, which is often associated with tumour-suppressive functions, might further contribute to its anti-proliferative effects. This dual-targeting capability—α1A-AR and ER—positions SIL as a uniquely promising candidate for repurposing. It aligns with the growing understanding that many drugs have polypharmacological profiles, and their therapeutic potential can be unlocked by exploring these off-target effects.

SIL inhibits proliferation and attenuates cell migration, a crucial step in the metastatic cascade. This effect is likely mediated through interference with integrin-mediated adhesion, as suggested by the observed downregulation of E-cadherin. E-cadherin is a well-characterised intercellular adhesion molecule that is a tumour suppressor in epithelial cancers. Its loss or downregulation is a hallmark of the epithelial-mesenchymal transition (EMT), which enhances invasiveness and metastasis [28, 29]. However, emerging evidence suggests that E-cadherin is not merely a passive adhesion molecule but also an active signalling modulator. Studies indicate that E-cadherin can transduce intracellular signals that promote cell proliferation and survival [30, 31]. This dual role complicates its function in cancer progression, as its loss may facilitate metastasis while its presence may sustain survival pathways in specific contexts. In this study, SIL treatment impaired cell-cell aggregation in 3D cultures of MCF-7 and MDA-MB-468, an effect that appears to be dependent on E-cadherin. Although E-cadherin is essential for maintaining epithelial integrity, its downregulation by SIL may explain the observed reduction in cohesive cluster formation, potentially contributing to a reduced metastatic potential.

Remarkably, SIL also reduced the ability of cancer cells to form stable spheroids, a hallmark of tumorigenic and drug-resistant cell populations. Given that spheroid formation is closely linked to tumorigenicity and metastatic potential, these results highlight SIL’s dual role in inhibiting proliferation and impairing the structural organisation of cancer cells, a crucial step in early tumour development and dissemination.

In conclusion, our findings establish SIL as a promising, multitargeted therapeutic candidate that may be utilized in an adjuvant setting, applicable across various breast cancer subtypes. Its ability to concurrently impair tumour proliferation, survival, and metastatic dissemination, coupled with its well-established safety profile to normal cells and cost advantages, supports its translational potential for both HR+ and HR− diseases (Fig. S5).

A key consideration is the pharmacological relevance of the concentrations used in vitro (10–50 µM), which are higher than the peak plasma levels achievable with the standard urological dose (~0.1–0.5 µM) [15]. These concentrations were chosen based on our dose-response and IC_50_ data, as well as precedents in repurposing α1-AR antagonists for oncology, and are common in initial in vitro proof-of-concept studies to elucidate mechanisms of action within a short timeframe. Our study establishes a baseline for SIL’s intrinsic anti-tumour activity.

Future work will include in vivo studies to determine if effective antitumor concentrations can be achieved with tolerable dosing regimens, potentially using localized delivery systems (e.g., nano-formulations) or combination therapies to enhance efficacy. Lower, clinically achievable doses of SIL might sensitize cells to other chemotherapeutic agents, as previously shown with gemcitabine and cisplatin in different cancers [20, 21]. The novel discovery of its potential as an ER modulator through in silico docking opens a new avenue for understanding its mechanism and optimizing its application. Future studies should also focus on validating the ER-SIL interaction using competitive binding assays and reporter gene assays to determine if SIL acts as an agonist, antagonist, or selective ER modulator (SERM).

These insights will be critical for advancing SIL toward clinical translation.

## Materials and methods

### Cell culture and treatments

The human breast cancer cell lines, MCF-7 (HR+) and the highly aggressive, triple-negative breast cancer cell line (HR-, HER2-) MDA-MB-231 and MDA-MB-468, were grown in DMEM/F-12 medium supplemented with 10% fetal bovine serum (FBS), 1% L-glutamine, and 1 mg/mL penicillin/streptomycin. The T47D (HR+) cell line was grown in RPMI-1640 medium supplemented with 10% FBS, 1% L-glutamine, and 1 mg/mL penicillin/streptomycin. The non-tumorigenic human breast epithelial cell line MCF-10A was grown in DMEM/F-12 medium supplemented with 5% FBS, 1% L-glutamine, and 1 mg/mL penicillin/streptomycin.

Normal human and breast cancer epithelial cell lines were purchased from the American Type Culture Collection (ATCC, USA) and used within 4 months after resuscitation of frozen aliquots (less than 30 passages). Cells were maintained at 37 °C in a humidified atmosphere with 5% CO_2_ in culture medium for no more than 20 passages. Every 4 months, cells were authenticated by short tandem repeat analysis (AmpFLSTR Profiler Plus PCR Amplification Kit, Applied Biosystems, Monza Brianza, Italy) at our Sequencing Core. Morphology, doubling times, and mycoplasma negativity (MycoAlert, Lonza, ThermoFisher Scientific, Milan, Italy) were tested monthly.

For the experiments, cells were plated in complete medium and, 24 hours later, treated in a serum-free medium for the indicated time, as described in the Results section.

Silodosin (SIL) was dissolved in sterile dimethylsulfoxide (DMSO), and a 4 mM stock solution was prepared and stored in aliquots at −20 °C. Working concentrations were diluted in the appropriate medium. The final concentration of DMSO in the cell cultures was equal to or less than 0.5% and was not toxic to the cells.

All chemicals were from Sigma/Merck (Darmstadt, Germany).

### Cell proliferation assay

Cell proliferation was measured in four breast cancer cell lines, MCF-7, MDA-MB-231, T47D, and MDA-MB-468, and in a non-tumorigenic, human breast epithelial cell line, MCF-10A. Cells were cultured in 96-well plates and treated with either vehicle (DMSO, control, Ctrl) or increasing SIL concentrations (ranging from 10 to 50 µM). Cell proliferation was measured using a spectrophotometric dye incorporation assay, Sulforhodamine B (SRB) [32], after 24, 48, or 72 h of treatment. IC_50_ were determined by interpolation from dose-response curves using GraphPad Prism 4 software.

### Clonogenic assay

MCF-7 and MDA-MB-231 cells (1000 cells/plate) were plated in growth medium in 6-well plates and, after 24 h, treated with SIL 30 or 50 µM. Medium and SIL treatment were renewed every 3 days. After 15 days, surviving colonies were fixed in 4% paraformaldehyde (PFA) for 30 min and stained with 0.5% crystal violet. Then, the colonies were photographed using an Olympus DP50 camera and counted using ImageJ software.

### Soft agar anchorage-independent growth assay

MCF-7 and MDA-MB-231 cells (10^4^ cells/well) were resuspended in growth medium, containing 0.35% agarose, and plated on a 0.7% agarose bottom layer in 6-well plates. Two days after plating, growth medium containing vehicle or treatments (SIL 30 or 50 µM) was added to the top layer and replaced every 3 days. After 21 days, 500 µL of MTT was added to each well and incubated at 37 °C for 4 h. Plates were kept at 4 °C overnight, and colonies greater than 50 µm in diameter from triplicate assays were counted under an inverted microscope (Olympus BX51).

### Cell cycle analysis

To evaluate the cell cycle, MCF-7 and MDA-MB-231 cells were plated in well plates at a density of 5 × 10⁵ cells/well and treated with either a vehicle alone or SIL at concentrations of 30 or 50 μM for 48 h. Flow cytometry was performed according to the protocol described in [29]. In brief, cells were harvested after a 3-min treatment with 0.025% trypsin at 37 °C. Subsequently, 10% FBS in PBS was added, and the cells were centrifuged, washed with PBS, and fixed in 70% cold ethanol. These harvesting conditions ensured a high yield of intact cells. Following fixation, the cells were washed with PBS, treated with RNase (100 μg/mL) for 15 min at 37 °C, and stained with a propidium iodide solution (10 μg/mL) in the dark for 30 min. The samples were analysed using a CytoFLEX Beckman flow cytometer (Beckman-Coulter, Milan, Italy), and acquisition data were obtained with CytExpert Beckman Coulter software (version 2.4). All chemicals used were from Sigma/Merck (Darmstadt, Germany).

### RNA extraction, reverse transcription, and qRT-PCR

Total RNA was extracted using Trizol, following the protocol described in our previous work [33]. The extracted RNA was treated with RNase-free DNase I to remove any contaminating DNA and then reverse-transcribed using the OneScript Plus cDNA Synthesis Kit with an oligo(dT) primer (ABM). Quantitative real-time PCR (qRT-PCR) was performed using an ABI PRISM 7000 Sequence Detection System (Applied Biosystems) with SYBR Green ReadyMix (Applied Biosystems) and gene-specific primers (details provided below). The GAPDH gene was used as an internal control to normalise the data. Gene expression levels were determined based on the threshold cycle (Ct) values, and relative expression was calculated using the 2^−ΔΔCt^ method, normalized to the expression of the housekeeping gene GAPDH.

The following primers for qRT-PCR were used:

Human Cyclin D1 for TCTAAGATGAAGGAGACCATC

Human Cyclin D1 rev GCGGTAGTAGGACAGGAAGTTGTT

Human Cyclin E for GAGGAAGAGGAAGATGAAGAGGA

Human Cyclin E rev GCTCCAGTTTGTTGATGGTCT

Human p53 for CAGCAGTCAGATCCTAGCGT

Human p53 rev TCATCCAAATACTCCACACGC

Human p21 for CCTGTCACTGTGTTGGCCTT

Human p21 rev GCAGAAGATGTAGAGCGGGC

Human p27 for TGGAGAAGCTAACCCGGAG

Human p27 rev GGTCTCTGCTCCACCAGTTC

Human E-cadherin for CCCACCACGTACAAGGGTC

Human E-cadherin rev CTGGGGTATTGGGGGCATC

Human α1A-AR for TTCAGCTTCATCGTCTTCTACC

Human α1A-AR rev GATGAAGATGGTGAAGTTGATGC

GAPDH for AGCCACATCGCTCAGACAC

GAPDH rev GCCCAATACGACCAAATCC

All reagents were from Invitrogen, Carlsbad, CA, USA.

### TUNEL assay

To measure DNA damage, MCF-7 and MDA-MB-231 cells were plated in growth medium for 24 h on coverslips in 35 mm Petri dishes at a density of 3 × 10^5^ cells/well, and then treated with either a vehicle alone or with 30 or 50 μM SIL for 72 h. Following the manufacturer’s instructions, apoptosis was assessed by enzymatically labelling DNA strand breaks using the DeadEnd Fluorometric TUNEL System (Promega, Milan, Italy). Coverslips were mounted on slides with Fluoromount mounting medium and examined under a fluorescence microscope (Olympus BX51). Nuclei were counterstained with DAPI. Apoptotic cells were imaged at 20X magnification using an Olympus DP50 camera and ViewFinder software, and the apoptotic index was quantified using ImageJ software. All chemicals were from Sigma/Merck (Darmstadt, Germany).

### Wound-healing scratch assay

MCF-7 and MDA-MB-231 cells were plated in growth medium in 6-well plates. After 2 days, confluence was reached, a scratch was made using a p10 tip, and the cells were treated with either a vehicle or SIL at 30 or 50 μM concentrations. After 48 h of treatment, the cells were fixed with 4% PFA and stained with 0.5% crystal violet. The pictures were taken at 10X magnification using a phase-contrast Olympus BX41 microscope equipped with CSV1.14 software and a CAMXC-30 camera. Images were captured immediately after the scratch (0 h) and 48 h later. The results shown are representative of three independent experiments, and the wound healing closure was quantified using ImageJ software.

### Spheroid‑forming assay

Spheroids were cultured following the protocol described in [34] in the appropriate Sphere Medium. MCF-7 and MDA-MB-468 cells were plated at a density of 4 × 10^5^ cells/mL in a single-cell suspension in 2% agar-coated plates and treated with either 30 or 50 μM of SIL for 48 h. To facilitate the formation of three-dimensional (3D) spheroids, the plates were rotated for 4 h at 37 °C. The 3D cultures were imaged using a phase-contrast microscope (Olympus, Milan, Italy). The degree of aggregation was quantified by measuring the spheroids with an ocular micrometre. Spheroids were categorised based on their smallest cross-sectional diameter into 25–50 μm, 50–100 μm, and >100 μm. Counts were performed across 10 different fields at 10X magnification.

### In silico molecular docking

Molecular docking simulations were performed to investigate the potential binding of SIL to human estrogen receptors (ER), starting from selected crystallographic structures of ERα and ERβ retrieved from the online Protein Data Bank (PDB): entry code 3ERT for ERα [35] and 2FSZ for ERβ [27]. Both structures are complexed with 4-hydroxytamoxifen (OHTX). 2FSZ also contains a secondary allosteric binding site occupied by an additional molecule of OHTX. The molecular structure of SIL was built and optimized using the modelling software Avogadro [36]. The ligand was prepared for docking in its fully protonated form at physiological pH. The conversion of the structure from the PDB format was performed by the graphic interface of AutoDockTools [37]. Docking calculations were performed by using AutoDock Vina [38]. During the conversion, polar hydrogens were added to the crystallographic receptor structure, whereas apolar hydrogens of the ligands were merged to the carbon atom they are attached to. Full flexibility was guaranteed for the ligand SIL, resulting in fifteen torsions. A single simulation run was carried out in each case at very high exhaustiveness, 16 times larger than the default value, to ensure comprehensive conformational sampling and reproducibility [39]. The docking grid was centred around the binding site coordinates of the co-crystallized ligand. For ERβ, separate simulations were performed at site 1 (the canonical binding pocket) and site 2 (a surface-exposed secondary site). Binding poses were ranked based on the predicted binding free energy (ΔG), and the top-ranked poses were analysed further. The binding modes of the ligand were analysed through visual simulations, and intermolecular interactions were evaluated using the protein-ligand intermolecular interaction profiler, PLIP [40].

Additionally, the inhibition constant (Ki) was calculated based on the binding energy according to the following equation:

$$\Delta {\rm{G}}={\rm{RT}}\mathrm{ln}({\rm{Ki}})$$

where ΔG, R, and T are related to the binding energy (kcal/mol), gas constant (1.987 cal/K mol), and temperature (310.15 K) [41, 42].

Finally, the ligand efficiency was calculated according to the following equation:

$$\mathrm{LE}=-{\Delta {\rm{G}}/N}_{\mathrm{non}-{\rm{H}}}$$

where *N*_non-H_ corresponds to the number of non-hydrogen atoms [43].

### Statistical analysis

Data were reported as mean values ± SD of at least three independent experiments. One-way or two-way analysis of variance (ANOVA) followed by Dunnett’s method was used to generate statistical analysis, as performed using the GraphPad Prism program; *p*-values < 0.05 were considered statistically significant.

### Ethics approval

Ethical approval was not required for this study as all experiments were performed on the cell lines and did not involve animals or humans.

## Supplementary information

Supplementary Figure Legends

Figure S1

Figure S2

Figure S3

Figure S4

Figure S5

## Data Availability

The authors declare that all data supporting the findings of this study are available in the article and its Supplementary Information files, or may be obtained from the corresponding author upon reasonable request.
