# Supplementary material for: Molecular insights into Silodosin’s anti-cancer effects: a promising repurposing strategy for breast cancer
Source: Cell Death Discov. 2026 Mar 5;12:128. doi: 10.1038/s41420-026-02973-8 (PMC13040017; doi:10.1038/s41420-026-02973-8)
Supplement: Supplementary file 1 — Supplementary Figure Legends [file 41420_2026_2973_MOESM1_ESM.docx]

Supplementary Information

Molecular insights into Silodosin’s anti-cancer effects: A promising repurposing strategy for breast cancer

Michele Pellegrino^1^, Maria Antonietta Occhiuzzi^1^, Maria Marra^1^, Francesca Giordano^1^, Fedora Grande^1^, Stefano Aquaro^2^, Stefania Marsico^1,§^ and Paola Tucci^1,§^

1. *Department of Pharmacy, Health and Nutritional Sciences, University of Calabria, 87036 Rende, Italy; michele.pellegrino@unical.it (MP); mariaantonietta.occhiuzzi@unical.it (MAO);* [*maria.marra@unical.it*](mailto:maria.marra@unical.it) *(MM); francesca.giordano@unical.it (FGi); fedora.grande@unical.it (FGr); stefania.marsico@unical.it (SM); paola.tucci@unical.it (PT)*
2. *Department of Life, Health and Environmental Sciences, University of L’Aquila, 67010 Aquila, Italy;* [*stefano.aquaro@univaq.it*](mailto:stefano.aquaro@univaq.it) *(SA)*

*§ Correspondence: stefania.marsico@unical.it (SM) and* [*paola.tucci@unical.it*](mailto:paola.tucci@unical.it) *(PT) Tel.: +39-0984493185*

**SUPPLEMENTARY FIGURE LEGENDS**

**Figure S1.** SIL does not affect normal breast cell growth. Non-tumorigenic human breast epithelial cell line MCF-10A was treated with increasing concentrations (0, 10, 20, 30, 40, 50 μM) of SIL for 24, 48, and 72 hours, as indicated, and cell viability was assessed by SRB assay. The results are expressed as % respect to control (Ctrl) cells. Data represent mean ± SD of three different experiments analysed in triplicate.

**Figure S2.** SIL inhibits breast cancer cell growth. Cells were treated with vehicle or 30 and 50 μM of SIL for 24, 48, and 72 hours, as indicated, and cell viability was assessed by SRB assay in (A) T47D, and (B) MDA-MB-468 cells. The results are expressed as % respect to control (Ctrl) cells. Data represent mean ± SD of three different experiments analysed in triplicate. *p<0.05, **p<0.01.

**Figure S3.** SIL inhibits the growth of breast cancer cells, sparing normal breast cells. (A) Cell survival curves across all tested cell lines at 24, 48, and 72 hours for the concentrations of 30 and 50 µM of SIL. The results are expressed as % respect to control (Ctrl) cells. Data represent mean ± SD of three different experiments analysed in triplicate. (B) SIL half-maximal inhibitory concentration (IC_50_) values for the indicated cancer cells were calculated at 48 hours using GraphPad Prism 4 (GraphPad Software).

**Figure S4.** The mRNA expression levels of α1A-AR were evaluated by qRT-PCR in all breast cancer cell lines used, compared to the non-tumorigenic MCF-10A cell line. The qRT-PCR results were normalized to the GAPDH gene. Data represent mean ± SD of three different experiments analysed in triplicate. *p<0.05, **p<0.01, ***p<0.001.

**Figure S5.** Graphical abstract that summarizes the main findings of our study: the effects of SIL on cell proliferation, cell cycle, apoptosis, migration, and spheroid formation in heterogeneous breast cancer cells (adapted from B. Firatligil-Yildirir, O. Yalcin-Ozuysal, N. Nonappa, Recent advances in lab-on-a-chip systems for breast cancer metastasis research, Nanoscale Adv 5 (2023) 2375–2393).
