# Supplementary figures and images for: Molecular insights into Silodosin’s anti-cancer effects: a promising repurposing strategy for breast cancer
Source: Cell Death Discov. 2026 Mar 5;12:128. doi: 10.1038/s41420-026-02973-8 (PMC13040017; doi:10.1038/s41420-026-02973-8)

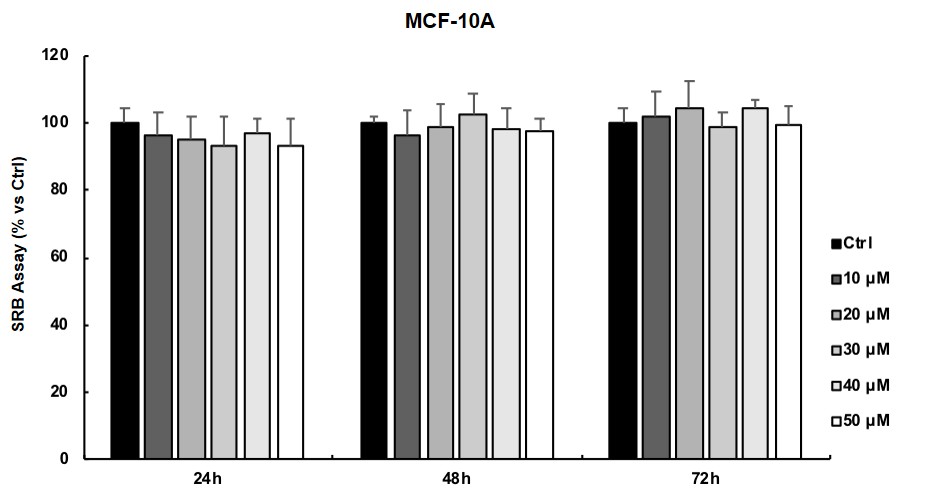

Supplement: Supplementary file 2 — Figure S1 [file 41420_2026_2973_MOESM2_ESM.jpg]

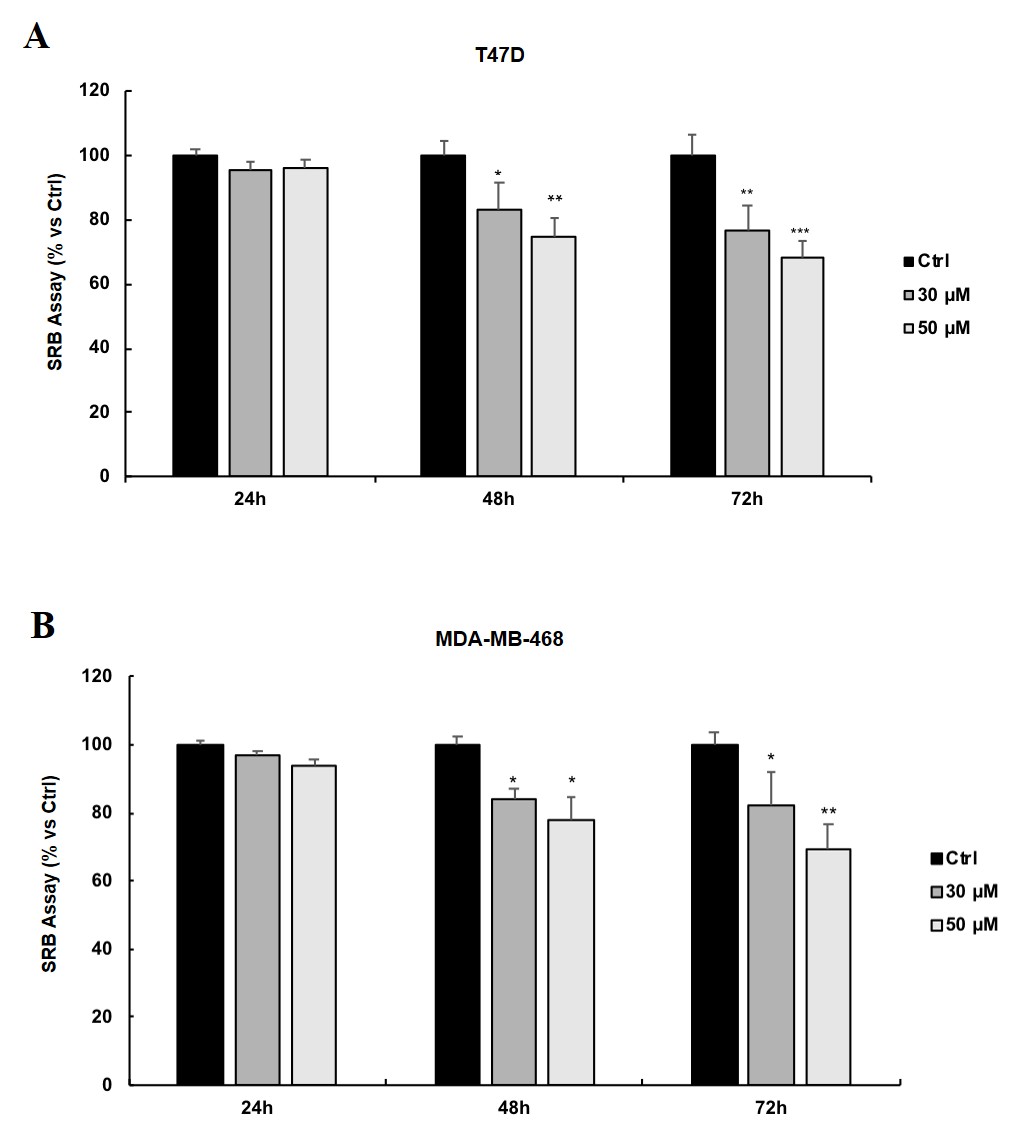

Supplement: Supplementary file 3 — Figure S2 [file 41420_2026_2973_MOESM3_ESM.jpg]

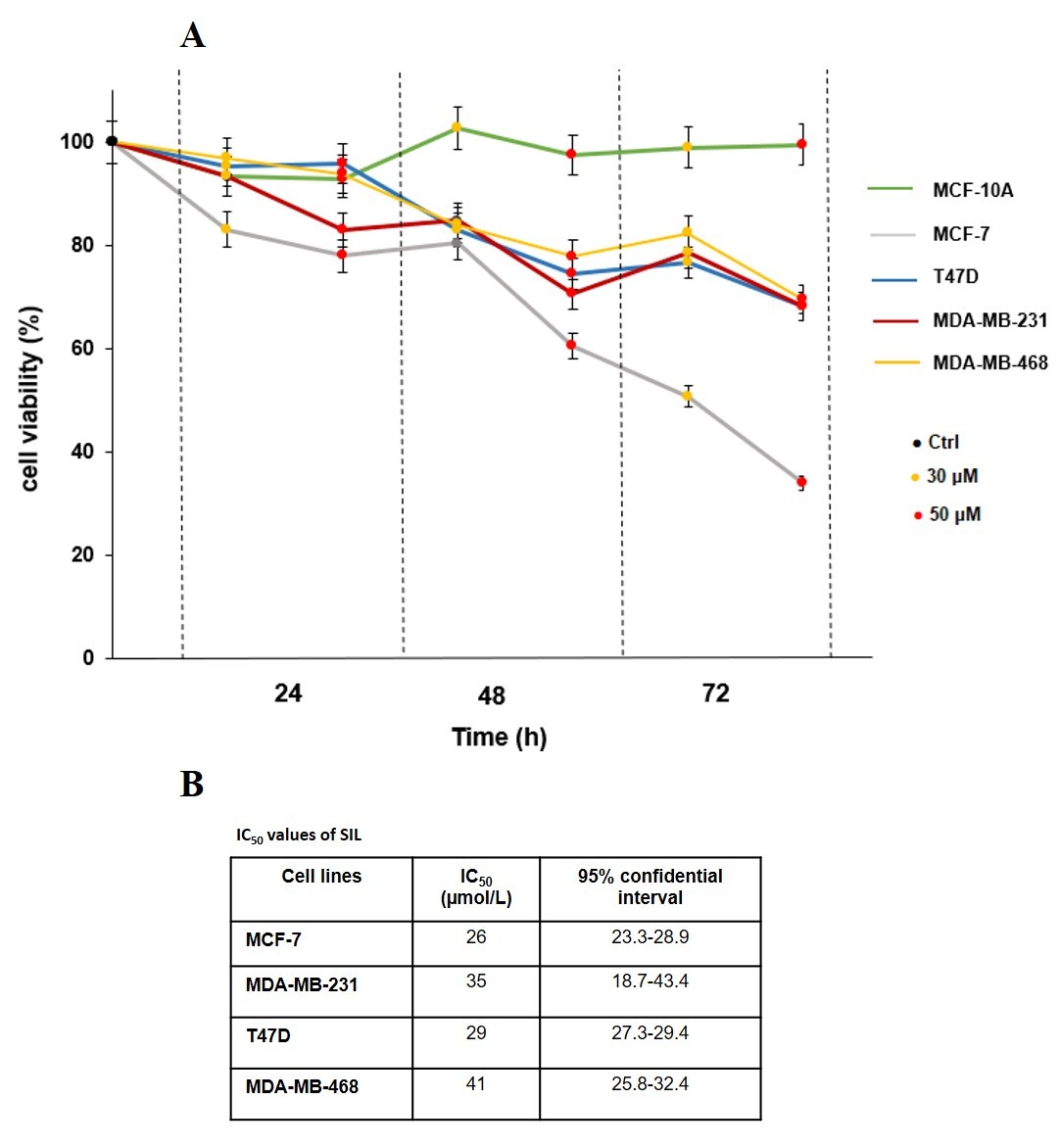

Supplement: Supplementary file 4 — Figure S3 [file 41420_2026_2973_MOESM4_ESM.jpg]

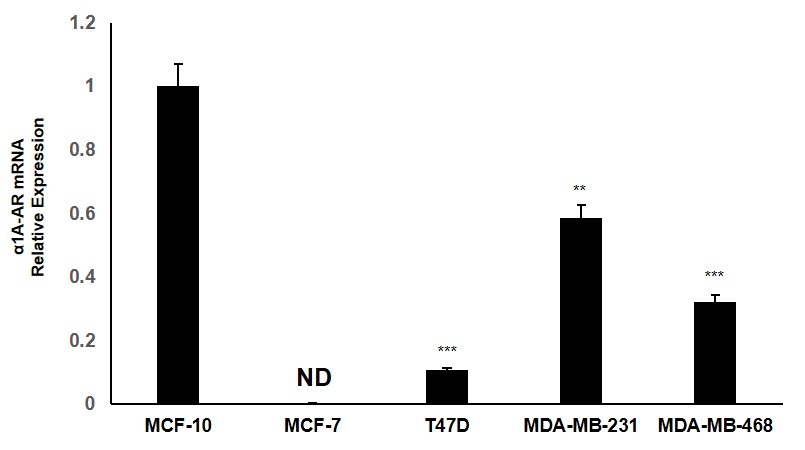

Supplement: Supplementary file 5 — Figure S4 [file 41420_2026_2973_MOESM5_ESM.jpg]

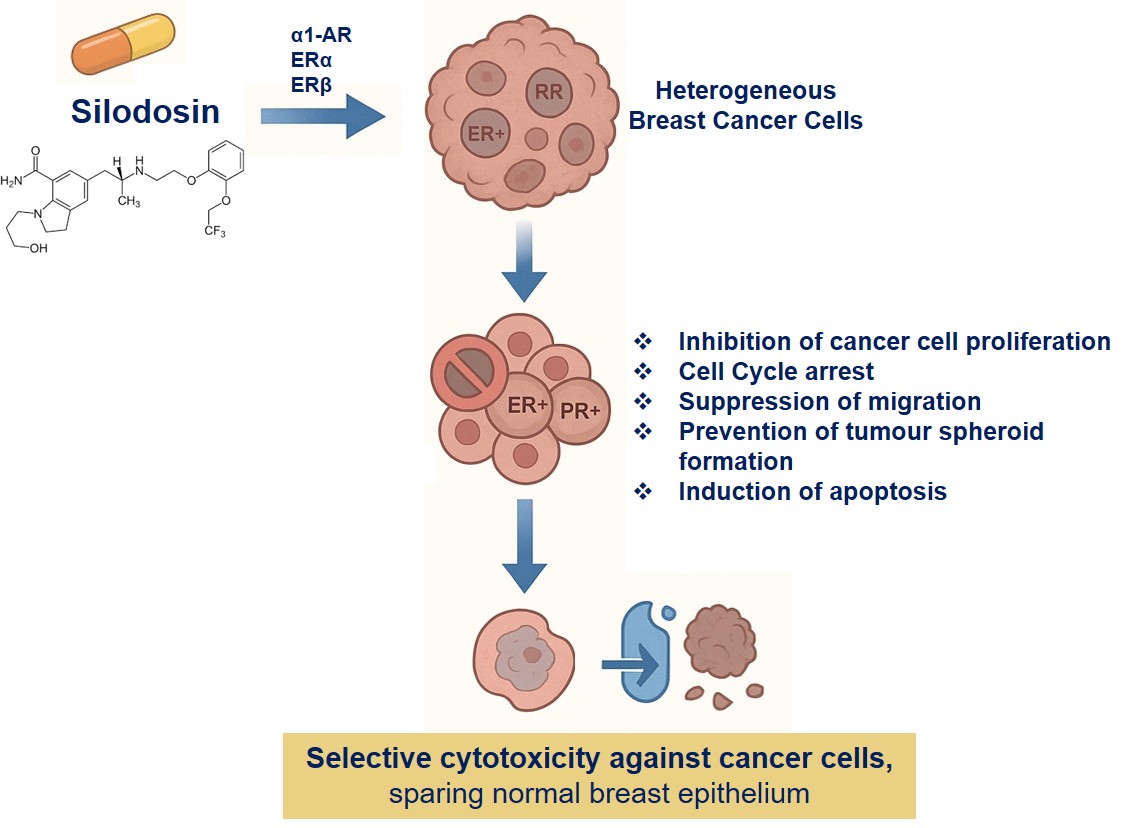

Supplement: Supplementary file 6 — Figure S5 [file 41420_2026_2973_MOESM6_ESM.jpg]
